# Genotypic Diversity and Genome-Wide Association Study of Protein Content and Amino Acid Profile in Diverse Potato Accessions

**DOI:** 10.3390/foods14122039

**Published:** 2025-06-09

**Authors:** Haroon Rasheed, Yining Ying, Daraz Ahmad, Bowen Deng, Jinsong Bao

**Affiliations:** 1 Institute of Nuclear Agricultural Sciences, Zhejiang University, Zijingang Campus, Hangzhou 310058, China; haroonzju@zju.edu.cn (H.R.); ying_erin@163.com (Y.Y.); darazahmad786@gmail.com (D.A.);; 2 Hainan Institute, Zhejiang University, Yazhou Bay Science and Technology City, Yazhou District, Sanya 572025, China

**Keywords:** potato, genotypic diversity, amino acid, nutritional quality, environment

## Abstract

The genotypic diversity and genome-wide association study (GWAS) of potato proteins and amino acid content were investigated in two environments: 98 potato accessions in Environment I and 93 in Environment II. Results revealed that aspartic acid was the most abundant amino acid in environment I and glutamic acid in environment II. The limiting amino acids were cysteine in both environments. The environmental variance accounted for more than 40% of the total variance for all traits except for serine and gamma amino butyric acid (GABA), indicating that potato protein and most amino acids were affected by growing seasons. GWAS identified 78 significant loci associated with potato protein and amino acid contents. The pleiotropic loci, especially those located on chromosomes 6, 9, and 11, provide a strong genetic basis for quality improvement. This study provides genetic insights into potato proteins and amino acid diversity, thereby enhancing molecular breeding for nutritional qualities.

## 1. Introduction

Potato (*Solanum tuberosum* L.) is the world’s most important non-cereal crop. It is not only a rich source of energy due to its high starch content [1], but also provides huge amounts of nutrients such as proteins, amino acids, vitamins, and minerals, which are beneficial to human health [2,3,4].

Potato proteins are known as allergy-free and are consumed by people of all ages, especially young babies who are allergic to other proteins [5]. Potato protein contains extensive amounts of both essential amino acids such as (valine, leucine, isoleucine, threonine, phenylalanine, lysine, histidine, and methionine) and non-essential amino acids such as (aspartic acid, glutamic acid, tyrosine, serine, glycine, alanine, cysteine, arginine, and proline) [6,7]. The most abundantly occurring amino acids are aspartic and glutamic [8]. Both are non-essential amino acids and play a role in many biological processes. Specifically, aspartic acid [9] and glutamic acid [10] inhibit the browning effects of fresh-cut potatoes by inhibiting polyphenol oxidase activity, thus regulating amino acid metabolism. Essential amino acids are crucial, as humans and other animals cannot synthesize them. The percentage of essential amino acids (47.9%) is closely comparable to the amount found in egg protein (49.7%) [7]. Lysine is an essential amino acid that is abundantly found in potato protein, distinguishing it as a better choice in comparison to maize, rice, and wheat [11,12,13]. Therefore, it is better to consume potatoes in combination with other limiting foods in lysine, such as rice, which will provide a good-quality protein [12]. Lysine plays a crucial role in the body’s absorption of calcium to produce collagen protein, a key component for maintaining strong bones and connective tissues [14]. On the other hand, potatoes contain a relatively lower level of sulfur-containing amino acids (methionine and cysteine) compared to other food crops [11,13], making it a limiting factor in the diet when measured against the daily intake values recommended by the World Health Organization [15]. As a limiting amino acid, methionine also plays a crucial role in influencing flavor in potatoes, as it is a precursor to methional, a key aroma compound in potato chips and fries [16,17]. That is why enhancing methionine levels in potatoes could provide nutritional and flavor benefits of interest to the consumers and the food industry. Various transgenic approaches have been used previously to increase methionine content in potatoes [16,18,19]. Apart from cysteine and methionine, threonine, tyrosine, lysine, proline, and histidine play a key role as important antioxidant amino acids that make potatoes nutritionally more stable and crucial for human health [6,11]. This nutritional profile demonstrated the significant contribution of potatoes to the natural world in promoting overall health. Therefore, enhancing protein and amino acid content is a desirable objective to improve the nutritional composition of potatoes [20]. However, the genetic basis of protein content and amino acids is not fully understood.

GWAS is the most powerful tool to identify markers associated with the desired traits [21]. Recent advancements in genome-wide association (GWAS) have identified genetic loci for various quality traits in potatoes [22,23]. Various quantitative trait loci (QTLs) have been discovered for various agronomic traits, specifically tuber yield, peel, and flesh color [24,25], flowering time, disease protection, temperature sensitivity [26], eye depth, tuber number, tuber shape, and weight [27,28]. A study identified some single nucleotide polymorphisms (SNPs) on chromosome 11 that are significantly associated with wart disease resistance [29]. A GWAS study identified QTLs on chromosomes 3, 5, 7, and 12 for protein content in 227 genotypes in various environments and years. This study identified a late maturity allele (*StCDF1)* on chromosome 5 strongly associated with protein contents [30]. A study detected 33 QTLs associated with 13 amino acids in 217 potato clones in various environmental conditions [31]. It is well known that the previously reported SNPs and candidate genes for various quality traits have led to significant advancements in potato biology; however, to the best of our knowledge, the genetic basis of potato protein and amino acids is not fully elucidated.

Thus, the objectives of the present study are: (1) to investigate the genotypic diversity in protein and amino acid content in various potato accessions in different environmental conditions; (2) to assess the genotype × environment interaction effects on potato protein and amino acids accumulation; and (3) to identify significant markers and candidate genes associated with these traits. The results of the current study may further contribute to our understanding of the protein and amino acid composition of potatoes and facilitate the selection and advancement of new breeding lines high in protein and amino acid contents through breeding programs.

## 2. Materials and Methods

### 2.1. Plant Materials and Preparation of Wholemeal Potato Powder

A diverse panel of 104 potato accessions was cultivated in the field of Huajiachi Campus at Zhejiang University in Hangzhou, China in 2019 (Environment I) and 2020 (Environment II) at the same location, employing a randomized complete block design with two replications (Table S1). The detailed methodology and environmental conditions were the same as previously reported [32]. After harvesting, potato tubers were cleaned, peeled, sliced, and freeze-dried using a freeze dryer (Boyikang Experimental Instrument, Beijing, China) at −50 °C for 72 h. The resultant freeze-dried potato pieces were then ground into a fine powder by using liquid nitrogen to avoid oxidation and potential contamination during grinding. Finally, the fine powder of 98 potato accessions in environment I and 93 in environment II were assessed for protein and amino acid content analysis. Of these, 89 common potato accessions in both environments were evaluated for genotype × environment interaction effects on protein and amino acid accumulation.

### 2.2. Determination of Protein Contents

The protein content was analyzed by using the standard protocol of the Kjeldahl method [33]. The potato fine powder (0.5 g) was digested in concentrated sulfuric acid for the conversion of organic nitrogen into ammonium sulfate. The mixture was diluted using a Kjeldahl distillation unit, and the ammonia was trapped in a boric acid solution. Each time, the mixture was distilled by using a Kjeldahl distillation unit, and the ammonia was subsequently suspended in a boric acid solution and titrated with standard HCl. Each time, the blank and standard samples with known nitrogen contents were analyzed. The potato protein was calculated by multiplying nitrogen content by a conversion factor of 6.25.

### 2.3. Determination of Amino Acid Contents

The amino acid contents were determined following the methods described by Zhong et al. [34,35] and Liyanaarachchi et al. [34,35] with minor modifications. Briefly, 0.2 g of potato fine powder was weighed and dissolved in 5 mL of 6 M HCl solution in a screw-capped tube. The air from the tubes was driven out for more than 10 sec using a rotary evaporator, and the caps were tightly closed. The mixture was then heated in an oven at 110 °C for 23 h. After 23 h, the mixture was cooled and transferred to a 50 mL round-bottom flask, and the volume was adjusted to 50 mL by adding ddH_2_O. Then, 1 mL of the diluted solution was moved to a separate 2 mL centrifuge tube. To dry the solution, the ddH_2_O and HCl were evaporated from the tubes using a nitrogen evaporator (12 Position N-EVAP Nitrogen Evaporator). After dryness, 2 mL of 0.02 M HCl was added to dilute the residue obtained, and then the solution was filtered with a 0.22 µm filter membrane into a separate bottle. About 20 µL of the final solution was injected into an auto-sampler bottle, and the analysis was performed using an amino acid auto-analyzer (L-8900 Hitachi Hight-Tech Corporation, Tokyo, Japan). The assay was conducted in duplicate for each sample, and the amount of amino acid was calculated in milligrams per gram (mg/g) dry weight of potato powder.

### 2.4. SNP Calling and Population Structure Analysis

SNP calling and population structure analysis were the same as previously reported in detail [32].

### 2.5. Genome-Wide Association Study

GWAS was conducted based on 226,487 SNPs and 22,115 insertion/deletions (InDels) as previously reported [32]. The primary principal component was included as the fixed covariate, and the genetic kinship as the random effect to the fixed-and-random model-circulating probability unification (FarmCPU) model in rMVP software (version 1.1.0) for association analysis [36]. The genome-wide significant threshold of the GWAS (*p*-value = 4.02 × 10^−6^) was determined by 1/*n* (*n* being the number of markers).

### 2.6. Statistical Analysis

Statistical analyses, including descriptive statistics (average, minimum, and maximum), and Pearson correlation, were conducted using IBM SPSS Statistics 27 (IBM Corp., Armonk, NY, USA). Analysis of variance (ANOVA) was conducted in a generalized linear model (GLM) using SAS (SAS Institute, Cary, NC, USA, version 9.4). Distribution analysis and normality tests based on the Shapiro–Wilk test [37], were conducted using JMP Pro 17 (SAS Institute Inc., Cary, NC, USA). Box plot analysis was performed using the ggplot2 package (version 2.2.0) in R studio, while principal component analysis (PCA) analysis was performed using Origin pro2023 (OriginLab. Corp., Northampton, MA, USA).

## 3. Results

### 3.1. Genotypic Diversity of Potato Protein and Amino Acids Profile in 2019

The genotypic diversity of 98 potato accessions in 2019, representing environment I, exhibited considerable phenotypic variation in protein and amino acid content. The average of total non-essential amino acids (56.1 mg/g) was higher compared to essential amino acids (25.6 mg/g) throughout the panel (Table 1). Across the panel, aspartic acid was the most abundant amino acid with an average of 19.8 mg/g. Glutamic acid was the second most abundant with an average of 18.5 mg/g, followed by lysine (4.84 mg/g), valine (4.56 mg/g), leucine (4.48 mg/g), arginine (4.32 mg/g), while cysteine was the least abundant amino acid with 0.69 mg/g of potatoes. The protein content ranged from 5.52 to 12.4%. The phenotypic distributions of protein and amino acid contents, as per the Shapiro–Wilk test (W), were normal except for a few deviations, indicating wide genotypic diversity of potato protein and amino acid profile (Figure S1A). This diversity suggests that potato genotypes in environment I exhibited a broad genetic diversity, providing opportunities for selecting high-performing varieties suited to food production and quality enhancement.

### 3.2. Genotypic Diversity of Potato Protein and Amino Acids Profile in 2020

In 2020 (environment II), the genotypic diversity of 93 potato accessions revealed a change in phenotypic performance due to slightly different environmental conditions. The average of total non-essential amino acids (70.4 mg/g) was higher compared to essential amino acids (28.9 mg/g) throughout the panel. Across the panel, glutamic acid was the most abundant amino acid with an average of 26.1 mg/g (Table 1). Aspartic acid was the second most abundant with an average of 23.3 mg/g, followed by arginine (6.17 mg/g), lysine (5.25 mg/g), valine (5.07 mg/g), leucine (5.00 mg/g), phenylalanine (4.21 mg/g), while cysteine was the least abundant amino acid with 0.76 mg/g of potatoes. The protein content ranged from 7.79 to 17.8%. The phenotypic distributions of protein and amino acid contents were broader than in 2019, indicating more variability in trait expression, possibly due to genotype-by-environment interaction (Figure S1B). This wide genotypic diversity of protein and amino acids highlighted potatoes’ resistance and potential for targeted breeding to address issues in food production under environmental fluctuations.

### 3.3. Genotype-by-Environment Interaction on Potato Protein and Amino Acids Diversity

**Figure 1:**
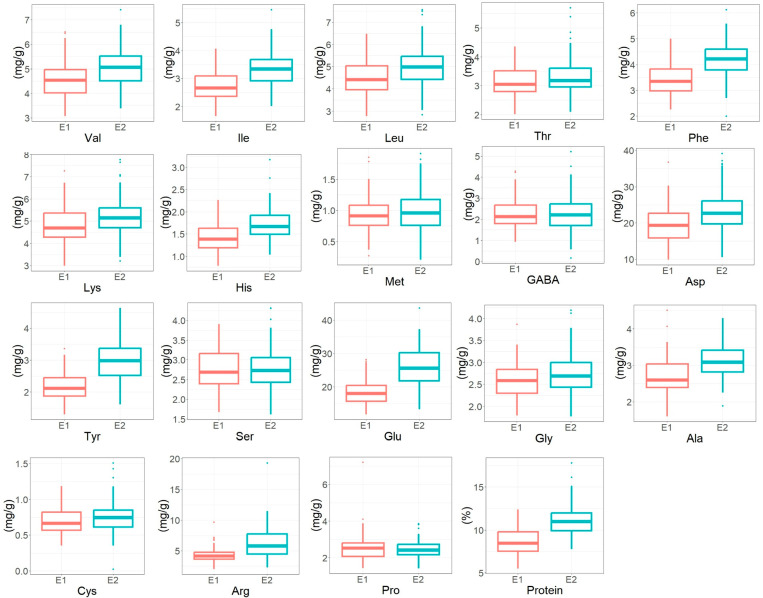
Diversity of potato protein and amino acids across two environments. E1, environment-I; E2, environment-II; result indicated amino acid as mg/g; protein in percentage.

Analysis of variance indicates that the protein content and amino acids were all affected by genotypic variance (G) except methionine (Table 2). This indicated that varietal differences existed broadly in the protein and amino acid levels in potatoes, as shown in Figure 1. Environmental effects (E) significantly affected the protein content and most amino acids, except for serine and GABA. The genotype-by-environment interaction (G × E) variance was significant for all the traits except for methionine and GABA. However, environmental variance accounted for more than 40% of the total variance for all traits except serine and GABA, indicating that most amino acids and proteins were mainly affected by environmental factors. Therefore, the overall finding demonstrated that environments mainly affected potato protein and amino acids.

### 3.4. Hierarchical Cluster Analysis

**Figure 2:**
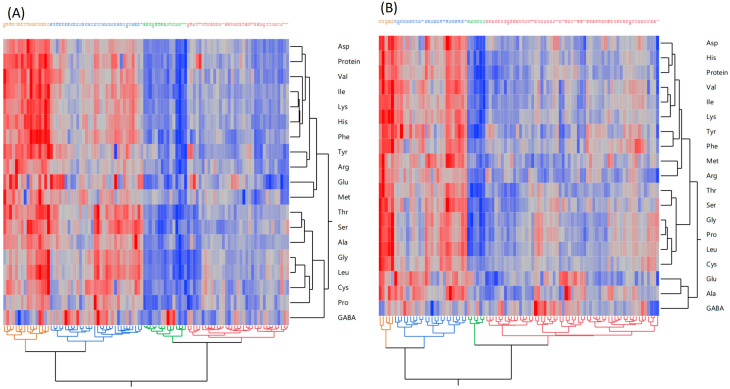
Protein and amino acid profile clustering in diverse potato accessions in two environments. Hierarchical clustering analysis based on the two-way Ward method. (**A**) represents Environment I and (**B**) Environment II. Each row in the heat map represents an individual amino acid and protein content, while the column represents different potato accessions. The color scale in the heat map represents the relative concentrations of amino acids, where the red color indicates a higher concentration, while the blue color indicates a lower level of protein and amino acid profile.

Hierarchical cluster analysis represented the relationship and the distinct pattern of all 18 amino acids and protein content between the two environments (Figure 2). The heat map classified moderate-lower, lower, moderate-higher, and highest amino acid and protein-containing accessions. The color intensity further demonstrated the superiority of environment II over environment I. The dendrogram adjacent to the heat map illustrated hierarchical relationships and clustering of potato protein and amino acids. In environment I, leucine and glycine were the most strongly associated amino acids, followed by lysine and isoleucine, and lysine and histidine (Figure 2A). In environment II, proline and glycine (Figure 2B) were the most strongly associated amino acids, followed by valine and isoleucine, suggesting potential biological significance.

### 3.5. Principal Component Analysis

**Figure 3:**
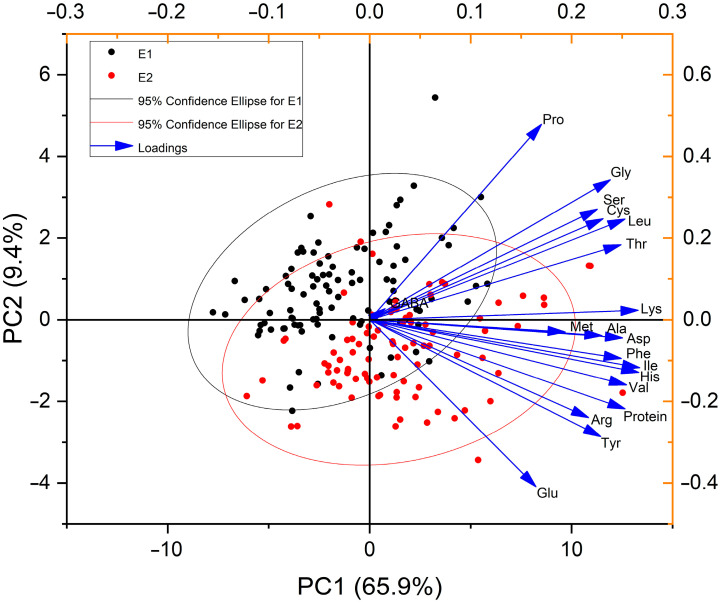
Principal component (PCA) visualization of protein and amino acid response to the diversity of environments. PCA separates potato accessions across two environments based on (mean of two repeats), protein, and amino acid composition in a diversity of potato accessions. Each point represents two different environments (Black, Environment I, and Red, Environment II). The ellipse represents 95% confidence intervals around the centroid of each data cluster.

Principal component analysis (PCA) was used to reduce dimensionality and identify patterns in large datasets. Most of the potato accessions associated with Environment II exhibited higher values (Figure 3). The first two principal components explain most of the variance in the data, accounting for 75.3% of the total variance, with the first principal component (PC1) explaining 65.9% and the second principal component (PC2) explaining 9.4% of the variance. All the tested amino acids and protein content clustered together in both environments, showing a strong positive correlation, except for GABA, which demonstrated no connection to any of the tested amino acids and protein content in the diverse panel of the potato accessions. Additionally, glutamic acid and proline were found at orthogonal angles, indicating no relationship with each other.

### 3.6. Correlation Analysis

The results of the Pearson correlation are shown in Table 3. Most amino acids were correlated positively in both environments. Specifically, glycine and leucine, valine and isoleucine, lysine and isoleucine, and leucine and proline exhibited a stronger positive correlation across both environments. The exceptions to this were GABA and glutamic acid. Glutamic acid showed no correlation to proline in both environments and phenylalanine in Environment II. In contrast to the other amino acids, GABA exhibited no correlations with any of the tested amino acids in both environments, except a positive correlation with glycine, leucine, proline, alanine, and cysteine in Environment I. Protein content showed the highest positive correlation with all the tested amino acids in both environments except with GABA. Moreover, most amino acids, including protein content, showed weak to non-significant correlations between the two environments (between the upper and lower diagonals) except threonine, glutamic acid, glycine, leucine, GABA, and arginine, which showed the highest positive correlations, indicating their strong interconnection between the two growing seasons.

### 3.7. Genome-Wide Association Study (GWAS) and Candidate Gene Analysis

**Figure 4:**
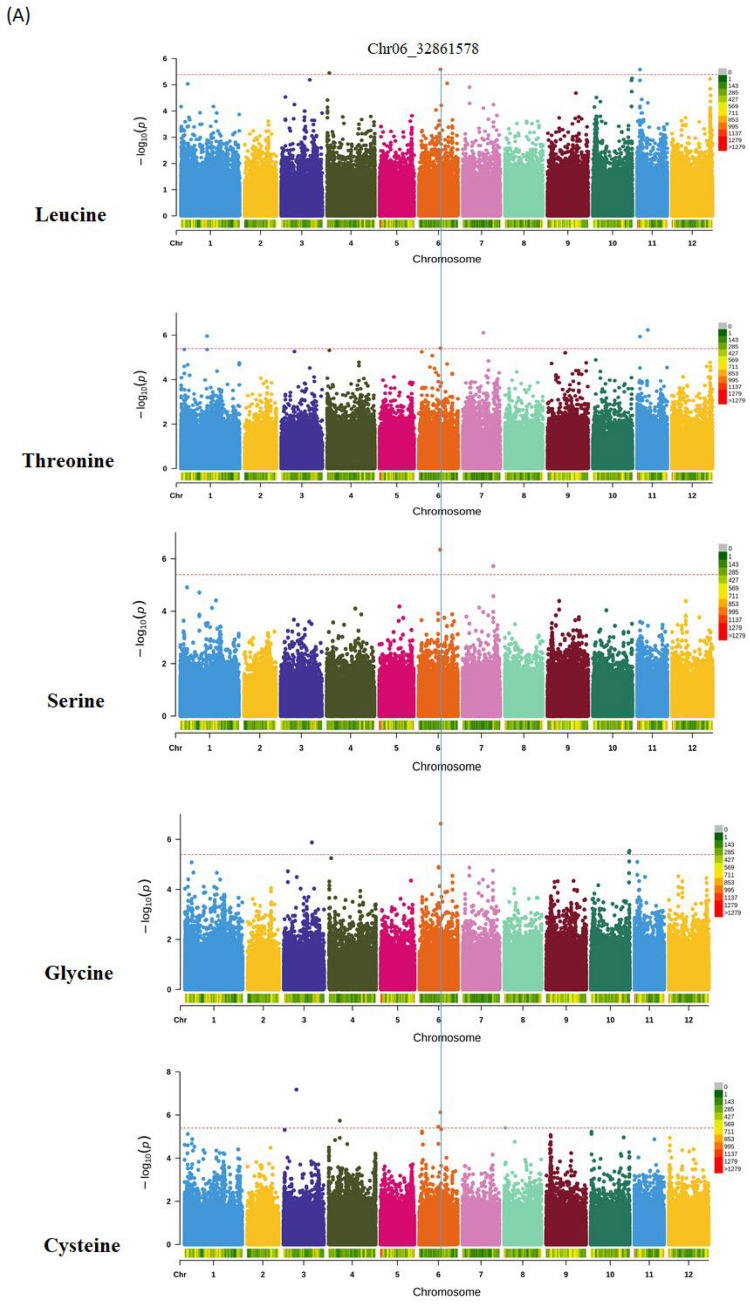

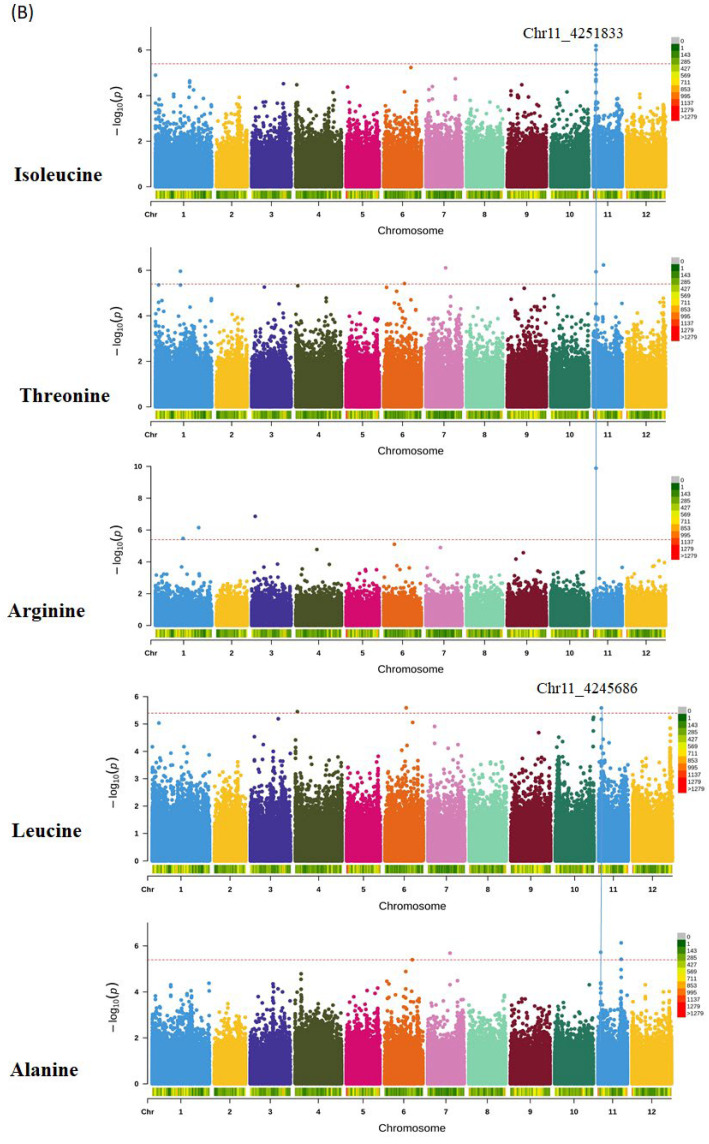
Genome-wide association study of potato protein and amino acid contents across two environments. (**A**) represents the pleiotropic gene on chromosome 6 (**B**) represents chromosome 11 with the pleiotropic gene. Each dot represents an SNP. The *x*-axis represents chromosomal numbers and SNP positions. The *y*-axis represents the negative logarithm *p*-value for individual SNPs. The broken red line represents the significant threshold 4.02 × 10^−6^. The heat maps at the bottom of each chromosome represent SNP density for potato protein contents and amino acids across two environments.

Manhattan plots visualized the GWAS of protein and amino acid profiles in a diverse panel of potato accessions across two seasons (Figure S2A–C). GWAS results for protein and various amino acid contents are presented in Table 4. A total of 48 significant SNPs or InDels in environment I and 30 in environment II were identified. These results indicated some important loci associated with most amino acid contents except valine, phenylalanine, aspartic acid, histidine, and protein in the environment I (Figure S2A–C; Table 4). Interestingly, the Manhattan skyline revealed chromosome 4 showing some unique features, but only touching the significant threshold for leucine in environment I. The most important chromosomes, 5, 6, 9, and 11, contributed 55% of the total significantly associated SNPs detected in the panel. Two interesting pleiotropic loci were found. One was on chromosome 6, responsible for leucine, threonine, serine, glycine, and cysteine (Figure 4A), and the other was on chromosome 11, responsible for isoleucine, threonine, arginine, leucine, and alanine (Figure 4B).

## 4. Discussion

### 4.1. Genotypic Diversity of Protein Content and Amino Acids in Potatoes

Genotypic diversity represents the overall variability present in an organism. It is essential to assess a diverse range of varieties for quality improvement. Enhancing protein and amino acid content in potatoes is a significant challenge in developing countries where plants constitute the primary protein source for humans and animals [38]. The current study revealed wide variations in the amounts of protein and 18 amino acids analyzed in a diverse collection of potato samples. Potatoes are more nutritionally valuable than cereals, although they have less protein [39], about 1 to 2% on fresh weight and 10% on a dry basis [40,41]. The same results were obtained in the current study, which revealed an average protein concentration of 9.95% across the diverse panels in two consecutive environments. The most abundant amino acids were aspartic acid and glutamic acid [8,42]. Similar results were obtained in the current investigations, ensuring the abundance of aspartic acid at 19.8 to 23.3 mg/g and glutamic acid at 18.5 to 26.1 mg/g in environments I and II, respectively. Aspartic and glutamic acids are the major amino acids, making up 47% of potatoes’ total amino acid content. The least abundant amino acids were methionine and cysteine, which agree with the previous studies [11,13]. Lysine and threonine are often found in lower concentrations in most plants, especially cereals; that is why proteins with high amounts of lysine are known as good supplements [43]. Lysine is an essential amino acid that promotes calcium absorption into the bones [14,42]. Potatoes have more lysine than other plant-based proteins [5,8]. It ranges from 1.10 to 5.56 mg/g [31], which is consistent with our current research findings (2.99 to 7.77 mg/g), but our results showed slightly higher amounts of lysine, which could be due to the use of high-quality accessions.

### 4.2. Effect of Environments on Protein Content and Amino Acids

Genotypic diversity assessment based on molecular characterization is more effective than morphological assessment, since it is greatly affected by the environment [39]. In this study, the growing seasons had a massive effect on the level of protein and amino acid content, resulting in significant variations between genotypes and the relative amount of protein and amino acids in the panel. Both essential and non-essential amino acids, along with protein content in environment II, were reported to be higher than in environment I. Especially, the abundance level of aspartic acid and glutamic acid fluctuated, with aspartic acid being the most abundant in environment I, and glutamic acid being the most abundant in environment II. Different potato genotypes may have different adaptations and stabilities over different environmental conditions. However, cysteine remained the least abundant, followed by methionine in both environments (Table 1). Based on the Pearson correlation, most of the amino acids exhibited stronger positive correlations across both environments, especially glycine and leucine, valine and isoleucine, lysine and isoleucine, and leucine and proline, indicating significant interconnection between the two consecutive environments. With changing environmental conditions, the production of cultivars that can tolerate fluctuations in the environment becomes more critical [44]. Therefore, various accessions showed consistency across the two environments. Specifically, SP71 was consistent among the top performers for potato protein contents, and key amino acids like glutamic acid, histidine, and arginine, SP33 for alanine, while SP19 for GABA across the environments. These accessions are strong candidates for potato quality improvement and breeding programs. However, it is obvious from ANOVA that most of the variations were due to genotype and environmental interaction, except methionine and GABA, which might be influenced by genetic factors (Table 2). These results prove that two environmental conditions play a role in influencing amino acids and proteins. Therefore, it is better to select varieties based on the improved protein and amino acid content by providing suitable environmental conditions for quality improvement.

### 4.3. Genetic Basis of Potato Protein Contents and Amino Acids

The significant association of phenotypic diversity for several traits with genetic markers can aid in understanding the genetic basis of protein and amino acids to improve health, nutritional qualities, and breeding efforts in potatoes. For the validation of current findings and to know the exact marker responsible for transferring these quality traits in potatoes, the SNP markers were generated using genome-wide association studies for 104 potato accessions based on the protein and amino acid contents. Results demonstrated that 78 SNPs were significantly associated with protein and amino acid content (Table 4). The significantly associated SNPs and candidate genes were detected for almost all the amino acids and proteins, excluding phenylalanine and histidine. The most abundant SNPs were detected for arginine, threonine, and GABA across the panel. Overall, GWAS results indicated that chromosomes 5, 6, 9, and 11 are the most important to evaluate potato protein and amino acid profiles. A study revealed that four significant loci were detected for potato protein contents on chromosomes 3, 5, 7, and 12. Where chromosomes 5 and 7 collectively explained 22% of the total variance [30], our study showed that chromosomes 5 and 9 were significantly associated with the protein contents in potatoes across the two environments. Two QTLs were reported for methionine on chromosomes 3 and 5 [45,46], but our results demonstrated five significant loci on chromosomes 5, 6, 8, 9, and 11. Levina et al. [47] detected two QTLs for arginine contents, while our results showed the most abundant SNPs for arginine contents on chromosomes 1, 3, 7, 8, 9, and 11 in the panel. There were no significant loci detected for tyrosine [46], while one significant locus was detected on chromosome 8 [48]. Our current study showed the same results, but the SNP was detected on chromosome 1. A recent study reported chromosomal positions and candidate genes for various amino acids, including a significant locus on chromosome 6 for histidine, and two loci for phenylalanine on chromosomes 2 and 10 [31], whereas the current study lacks SNP data for both amino acids in the whole panel. So, this study provides valuable genomic and metabolic insights that further our understanding of protein and amino acid diversity in potatoes. Previous studies found no SNP for methionine levels, but its level must be increased to meet the requirements of the potato industry, mainly due to its strong association with fragrance and the health-beneficial properties of potatoes [19,31], whereas 5 significant SNPs on chromosomes 5, 6, 8, 9, and 11 were detected in the current study. These novel findings provide the potential for targeted genetic modifications in protein and amino acid levels for potato quality improvement.

Some potential candidate genes were detected in the panel (https://phytozome-next.jgi.doe.gov/, accessed on 25 December 2023). The C-T substitution at Chr11_4251833 downstream of the gene (PGSC0003DMG400027362) was significantly associated with isoleucine, threonine, and arginine (Figure 4B). The variation is unlikely to alter the protein functions but might play a crucial role in quality improvement through further studies. Based on the KEGG pathway (SOT 102,578,720), ascorbate peroxidase enzyme PGSC0003DMG401001731 was mapped for GABA. Although it is not directly involved in amino acid metabolism, it plays a key role in the oxidative stress response that indirectly affects metabolic activities, including amino acid metabolism [49,50]. It uses ascorbate as an electron donor to convert H_2_O_2_ to H_2_O in chloroplasts, cytosol, mitochondria, peroxisomes, and the apoplastic space, thereby reducing the oxidative stress response [51]. Such associations with known functions indicated some evidence for the selection of desired traits. Therefore, it can be stated that the candidate gene analyses conducted have provided the genetic factors influencing protein and various amino acids, providing novel information for quality improvement in potato varieties. Another candidate gene PGSC0003DMG400013863, detected on chromosome 6, was associated with various amino acids such as leucine, threonine, serine, glycine, and cysteine (Figure 4A). Based on functional annotation, this gene might play an important role in many physiological processes by targeting serine or threonine residues [52]. Thus, the SNPs and candidate genes, especially those exhibiting pleiotropy, associated with various quality traits, have the potential to improve numerous desired characteristics in potatoes together and therefore need to be further studied.

## 5. Conclusions

Genotypic diversity and genome-wide association of potato protein and amino acids under two environmental conditions were investigated. The potato protein and amino acid contents were significantly affected by the growing seasons. The SNPs and candidate genes in the panel provided strong evidence of the genetic architecture of potato protein and amino acid profile, thereby offering novel information to breeding programs and marker-assisted selection for nutritional quality improvement.

## Figures and Tables

**Table 1:** Protein and amino acid contents across diverse potato accessions in two consecutive growing seasons.

	Environment-I			Environment-II		
Traits	Mean	Min.	Max.	Mean	Min.	Max.
Essential amino acids						
Valine	4.56	3.07	6.50	5.07 **	3.38	7.41
Isoleucine	2.74	1.66	4.06	3.38 **	2.02	5.45
Leucine	4.48	2.78	6.48	5.00	2.84	7.56
Threonine	3.14	2.02	4.35	3.31 **	2.10	5.69
Phenylalanine	3.44	2.26	5.00	4.21	2.00	6.13
Lysine	4.84	2.99	7.27	5.25 **	3.21	7.77
Histidine	1.43	0.79	2.27	1.74 **	1.04	3.17
Methionine	0.93	0.27	1.85	0.98 **	0.21	1.91
Total EAA	25.6	15.8	37.8	28.9 **	16.8	45.1
Non-Essential amino acids						
Aspartic acid	19.8	9.86	36.8	23.3 **	10.5	39.1
Tyrosine	2.19	1.30	3.36	2.95 **	1.61	4.63
Serine	2.75	1.67	3.91	2.78 *	1.62	4.31
Glutamic acid	18.5	11.7	28.2	26.1 **	13.3	43.6
Glycine	2.59	1.79	3.87	2.76	1.77	4.19
Alanine	2.72	1.60	4.51	3.12 **	1.89	4.29
Cysteine	0.69	0.35	1.18	0.76 **	0.02	1.51
Arginine	4.32	2.01	9.70	6.17	2.27	19.3
Proline	2.57	1.44	7.20	2.47 **	1.43	3.86
Total NEAA	56.1	31.8	98.7	70.4 **	34.4	124.8
GABA	2.32	0.94	4.30	2.28 **	0.17	5.21
Protein content (%)	8.70	5.52	12.4	11.2 **	7.79	17.8

Results are shown in milligrams per gram of dry weight (mg/g DW) of potato fine powder, Min., minimum; Max., maximum; Asp, aspartic acid, Thr, threonine; Ser, serine; Glu, glutamic acid; Gly, glycine; Ala, alanine; Cys, cysteine; Val, valine; Met, methionine; Ile, Isoleucine; Leu, leucine; Tyr, tyrosine; Phe, phenylalanine; GABA, gamma amino butyric acid; Lys, lysine; His, histidine; Arg, arginine; Pro, proline. * and ** indicate significant difference between two environments at *p* < 0.05, 0.01, respectively.

**Table 2:** Mean square values for protein and amino acid profile in potatoes.

Source	df	Asp	Thr	Ser	Glu	Gly	Ala	Cys	Val	Met
Genotype	89	41.2 **	0.59 **	0.34 **	39.2 **	0.31 **	0.34 **	0.05 **	1.02 **	0.08
Environment	1	689.9 **	1.59 **	0.12	2882.8 **	1.39 **	8.71 **	0.26 **	14.6 **	0.22 *
G × E	89	25.0 **	0.26 **	0.24 **	16.1 **	0.14 **	0.19 **	0.04 **	0.52 **	0.09
**Source**	**Ile**	**Leu**	**Tyr**	**Phe**	**GABA**	**Lys**	**His**	**Arg**	**Pro**	**Protein**
Genotype	0.51 **	1.17 **	0.43 **	0.61 **	0.98 **	1.13 **	0.17 **	6.24 **	0.50 **	4.29 **
Environment	21.1 **	14.2 **	29.7 **	29.9 **	0.02	10.5 **	4.95 **	171.7 **	0.61 *	296.8 **
G × E	0.28 **	0.59 **	0.27 **	0.42 **	0.30	0.63 **	0.11 **	2.36 **	0.37 **	2.67 **

* *p* < 0.05, ** *p* < 0.01, df, degree of freedom; G × E, genotype and environmental interactions.

**Table 3:** Pearson correlation amongst protein and 18 amino acid content across diverse potato samples in two consecutive environments.

Traits	Asp	Thr	Ser	Glu	Gly	Ala	Cys	Val	Met	Ile	Leu	Tyr	Phe	GABA	Lys	His	Arg	Pro	Protein
Asp	**0.19**	0.75 **	0.72 **	0.35 **	0.69 **	0.50 **	0.69 **	0.79 **	0.66 **	0.81 **	0.76 **	0.68 **	0.67 **	0.002	0.82 **	0.87 **	0.61 **	0.65 **	0.83 **
Thr	0.73 **	0.37 **	0.85 **	0.34 **	0.85 **	0.56 **	0.78 **	0.75 **	0.64 **	0.80 **	0.87 **	0.66 **	0.66 **	−0.09	0.81 **	0.80 **	0.69 **	0.80 **	0.81 **
Ser	0.69 **	0.87 **	**0.16**	0.37 **	0.82 **	0.61 **	0.75 **	0.69 **	0.68 **	0.76 **	0.83 **	0.58 **	0.63 **	−0.10	0.77 **	0.77 **	0.52 **	0.79 **	0.80 **
Glu	0.51 **	0.49 **	0.36 **	0.39 **	0.23 *	0.50 **	0.37 **	0.44 **	0.37 **	0.35 **	0.22 *	0.29 **	0.18	0.16	0.40 **	0.47 **	0.35 **	0.20	0.64 **
Gly	0.68 **	0.86 **	0.80 **	0.29 **	0.34 **	0.56 **	0.89 **	0.63 **	0.61 **	0.75 **	0.96 **	0.52 **	0.63 **	−0.07	0.79 **	0.70 **	0.51 **	0.96 **	0.70 **
Ala	0.75 **	0.82 **	0.81 **	0.50 **	0.75 **	0.26 *	0.57 **	0.68 **	0.60 **	0.67 **	0.61 **	0.49 **	0.60 **	0.15	0.65 **	0.57 **	0.38 **	0.47 **	0.63 **
Cys	0.63 **	0.69 **	0.62 **	0.25 *	0.85 **	0.63 **	**0.14**	0.68 **	0.70 **	0.78 **	0.89 **	0.59 **	0.66 **	−0.06	0.82 **	0.74 **	0.61 **	0.83 **	0.74 **
Val	0.82 **	0.75 **	0.67 **	0.68 **	0.57 **	0.77 **	0.52 **	0.26 *	0.65 **	0.94 **	0.74 **	0.82 **	0.82 **	0.12	0.88 **	0.88 **	0.68 **	0.57 **	0.83 **
Met	0.52 **	0.54 **	0.44 **	0.43 **	0.39 **	0.53 **	0.48 **	0.63 **	**−0.03**	0.68 **	0.64 **	0.58 **	0.61 **	−0.18	0.65 **	0.70 **	0.65 **	0.50 **	0.71 **
Ile	0.83 **	0.85 **	0.77 **	0.56 **	0.76 **	0.83 **	0.67 **	0.92 **	0.62 **	0.23 *	0.84 **	0.80 **	0.85 **	0.07	0.91 **	0.88 **	0.67 **	0.69 **	0.81 **
Leu	0.68 **	0.87 **	0.79 **	0.29 **	0.97 **	0.79 **	0.81 **	0.58 **	0.47 **	0.79 **	0.29 **	0.63 **	0.75 **	−0.04	0.86 **	0.77 **	0.56 **	0.93 **	0.75 **
Tyr	0.75 **	0.67 **	0.61 **	0.47 **	0.49 **	0.65 **	0.51 **	0.80 **	0.62 **	0.77 **	0.50 **	**0.15**	0.81 **	−0.01	0.79 **	0.75 **	0.63 **	0.44 **	0.66 **
Phe	0.83 **	0.83 **	0.79 **	0.43 **	0.80 **	0.79 **	0.68 **	0.82 **	0.59 **	0.91 **	0.81 **	0.75 **	**0.13**	0.02	0.80 **	0.69 **	0.53 **	0.55 **	0.62 **
GABA	0.12	0.20	0.17	0.07	0.21 *	0.26 **	0.27 **	0.16	0.20	0.16	0.20 *	0.14	0.13	0.55 **	0.03	0.03	−0.09	−0.05	−0.04
Lys	0.89 **	0.84 **	0.78 **	0.52 **	0.77 **	0.83 **	0.66 **	0.88 **	0.56 **	0.93 **	0.78 **	0.78 **	0.93 **	0.16	0.24 *	0.89 **	0.69 **	0.72 **	0.84 **
His	0.90 **	0.77 **	0.71 **	0.54 **	0.69 **	0.79 **	0.61 **	0.88 **	0.58 **	0.91 **	0.72 **	0.79 **	0.90 **	0.12	0.93 **	**0.13**	0.78 **	0.68 **	0.91 **
Arg	0.73 **	0.66 **	0.52 **	0.54 **	0.51 **	0.64 **	0.46 **	0.74 **	0.54 **	0.72 **	0.53 **	0.72 **	0.68 **	−0.02	0.75 **	0.79 **	0.53 **	0.48 **	0.72 **
Pro	0.58 **	0.60 **	0.60 **	0.18	0.74 **	0.57 **	0.67 **	0.43 **	0.24 *	0.53 **	0.70 **	0.30 **	0.59 **	0.21 *	0.59 **	0.54 **	0.35 **	**0.15**	0.68 **
Protein	0.86 **	0.73 **	0.64 **	0.63 **	0.60 **	0.72 **	0.57 **	0.84 **	0.54 **	0.82 **	0.60 **	0.69 **	0.78 **	0.16	0.86 **	0.86 **	0.76 **	0.53 **	**0.19**

Significant * *p* < 0.05, ** *p* < 0.01; lower diagonal represents environment I; upper diagonal represents environment II; value in bold in between the two diagonals represents the correlation between two environments.

**Table 4:** Key marker-trait associations in GWAS for protein content and amino acid profile across two seasons.

Traits	Env	Chr	Pos	Polymorphism	Effect	*p*-Value
Val	E2	3	30,733,104	G/T	0.96	3.63 × 10^−6^
	E2	5	29,423,795	A/G	0.94	2.74 × 10^−6^
	E2	7	16,612,597	T/G	1.02	6.00 × 10^−7^
	E2	8	8,200,367	T/A	1.00	3.93 × 10^−6^
	E2	10	11,259,467	A/T	1.05	9.13 × 10^−7^
	E2	10	42,742,706	G/A	0.93	3.62 × 10^−6^
	E2	12	46,651,522	T/A	1.07	7.08 × 10^−7^
Ile	E1	11	4,251,833	C/T	−0.62	6.45 × 10^−7^
Leu	E1	4	3,239,607	G/A	0.93	3.54 × 10^−6^
	E1	6	32,861,578	G/A	1.07	2.57 × 10^−6^
	E1	11	4,245686	A/G	−0.97	2.60 × 10^−6^
Thr	E1	1	39,366,436	A/T	0.62	1.10 × 10^−6^
	E1	6	32,861,578	G/A	0.70	3.82 × 10^−6^
	E1	7	31,045,779	A/G	0.67	7.82 × 10^−7^
	E1	11	4,251,833	C/T	−0.63	1.16 × 10^−6^
	E2	1	27,557,136	G/A	0.89	3.17 × 10^−6^
	E2	5	16,789,000	G/A	0.95	1.75 × 10^−6^
	E2	7	22,826,824	T/C	0.84	2.90 × 10^−6^
	E2	9	17,445,526	T/C	1.02	9.33 × 10^−7^
	E2	9	39,339,902	C/T	1.13	7.08 × 10^−7^
	E2	11	15,170,078	A/T	0.87	1.02 × 10^−6^
Lys	E1	11	38,266,005	G/A	1.13	2.30 × 10^−6^
Met	E1	5	30,125,638	A/C	0.36	1.89 × 10^−6^
	E1	8	13,060,996	A/G	0.36	3.14 × 10^−6^
	E1	9	5,875,273	G/T	0.41	2.96 × 10^−6^
	E1	11	44,175,166	T/C	0.30	3.40 × 10^−6^
	E2	6	40,534,805	C/A	0.46	4.92 × 10^−8^
Asp	E2	5	37,361,991	G/C	7.08	3.64 × 10^−6^
Tyr	E1	4	294,768	G/T	0.62	3.06 × 10^−6^
Ser	E1	6	32,861,578	G/A	0.72	4.49 × 10^−7^
	E1	7	46,305,291	A/G	0.68	1.90 × 10^−6^
	E2	9	39,339,902	C/T	0.86	3.60 × 10^−6^
Glu	E1	5	39,242,513	C/G	2.30	6.82 × 10^−8^
	E1	6	25,501,358	T/A	4.56	1.22 × 10^−15^
	E1	7	4,581,613	T/C	−1.99	1.95 × 10^−6^
	E1	7	34,579,854	A/G	−2.39	1.19 × 10^−8^
	E1	7	50,675,253	T/C	3.52	5.62 × 10^−12^
	E1	10	33,124,500	A/G	6.29	1.31 × 10^−29^
	E1	12	43,292,118	C/A	−2.23	1.95 × 10^−7^
	E1	12	45,133,407	C/A	−3.54	1.64 × 10^−9^
Gly	E1	3	43,175,911	C/A	0.50	1.33 × 10^−6^
	E1	6	32,861,578	G/A	0.57	2.37 × 10^−7^
	E1	10	58,454,528	A/T	0.59	3.32 × 10^−6^
	E1	10	59,143,531	T/TA	0.57	2.90 × 10^−6^
Ala	E1	6	42,964,473	A/G	0.59	3.97 × 10^−6^
	E1	7	34,678,031	A/T	0.79	2.06 × 10^−6^
	E1	11	4,245,686	A/G	−0.62	1.89 × 10^−6^
	E1	11	35,966,739	G/A	0.76	7.39 × 10^−7^
	E1	11	36,000,195	G/A	0.69	3.80 × 10^−6^
Cys	E1	3	19,968,173	A/G	0.21	6.63 × 10^−8^
	E1	4	17,072,661	T/C	0.23	1.82 × 10^−6^
	E1	6	29,637,957	G/A	0.22	3.45 × 10^−6^
	E1	6	32,861,578	G/A	0.22	7.46 × 10^−7^
	E1	8	1,880,790	T/C	0.20	3.92 × 10^−6^
	E2	9	42,997,248	G/T	0.25	3.27 × 10^−6^
Arg	E1	1	43,629,349	T/G	0.66	3.40 × 10^−6^
	E1	1	68,183,425	C/T	0.60	7.08 × 10^−7^
	E1	3	5,618,996	G/A	0.78	1.38 × 10^−7^
	E1	11	4,251,833	C/T	−0.85	1.30 × 10^−10^
	E2	1	8,356,1178	C/CTCGGAGAG	6.91	3.13 × 10^−6^
	E2	3	50,092	C/T	3.77	1.54 × 10^−6^
	E2	3	50,723,334	C/T	6.53	1.09 × 10^−6^
	E2	7	22,826,824	T/C	3.08	1.75 × 10^−6^
	E2	8	5,211,660	G/A	3.87	3.26 × 10^−6^
	E2	9	6,548,662	G/A	5.20	1.90 × 10^−6^
Pro	E1	5	2,070,162	G/A	2.77	3.00 × 10^−7^
GABA	E1	5	18,301,550	A/T	1.12	9.60 × 10^−8^
	E1	6	32,053,243	A/C	0.92	3.47 × 10^−6^
	E1	8	12,848,702	T/A	0.96	2.97 × 10^−6^
	E1	8	48,733,124	A/T	1.19	5.57 × 10^−7^
	E1	12	54,214,307	G/A	1.17	4.99 × 10^−7^
	E2	6	36,283,158	T/A	1.26	3.85 × 10^−6^
	E2	6	53,447,217	T/G	−1.42	2.43 × 10^−6^
	E2	9	6,605,261	A/G	1.13	1.79 × 10^−6^
	E2	9	56,125,912	A/G	1.25	1.80 × 10^−6^
	E2	12	6,262,001	G/T	−1.06	4.01 × 10^−6^
Protein	E2	5	2,601,172	G/A	2.32	3.50 × 10^−6^
	E2	9	58,640,876	A/T	2.52	1.94 × 10^−6^

Env, environment; E1, environment I; E2, environment II; Chr, chromosome; Pos, position.

## Data Availability

The original contributions presented in the study are included in the article/Supplementary Materials. Further inquiries can be directed to the corresponding author.

## Supplementary Materials

The following supporting information can be downloaded at: https://www.mdpi.com/article/10.3390/foods14122039/s1, Table S1: List of potato accessions; Figure S1: Distributions of protein and amino acids across diverse potato accessions in two environments; Figure S2: Genome-wide association study of protein and amino acid contents in potatoes across two environments.
