# Supplementary material for: Genotypic Diversity and Genome-Wide Association Study of Protein Content and Amino Acid Profile in Diverse Potato Accessions
Source: Foods. 2025 Jun 9;14(12):2039. doi: 10.3390/foods14122039 (PMC12192551; doi:10.3390/foods14122039)
Supplement: Supplementary file 1 [file foods-14-02039-s001.zip › foods-3664018-supplementary.pdf]

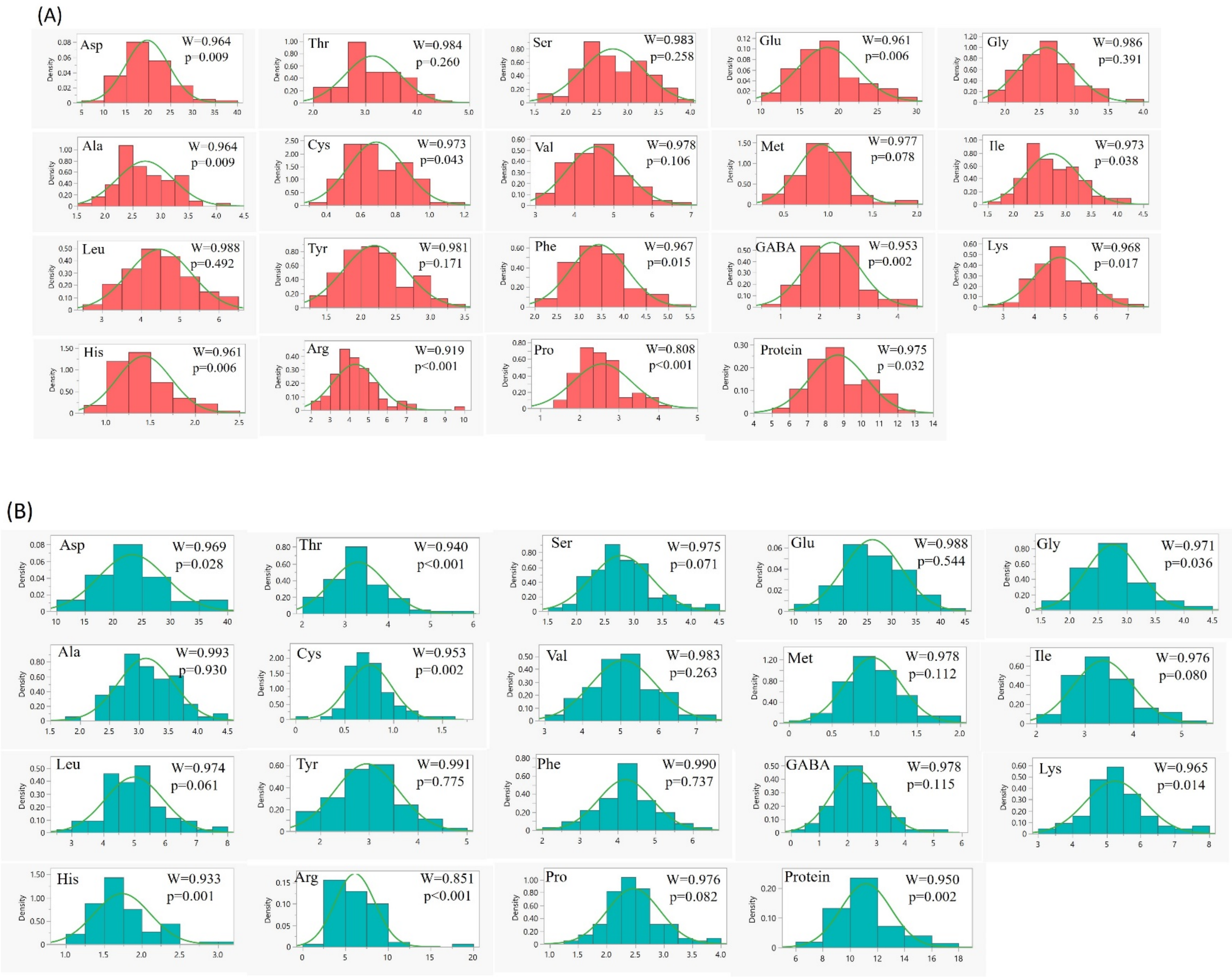

Figure S1. Distributions of protein and amino acids across diverse potato accessions in two environments. **(A)** represents environment I. (**B)** represents environment II; significant at $p<0.05$; Asp, aspartic acid; Thr, threonine; Ser, serine; Glu, glutamic acid; Gly, glycine; Ala, alanine; Cys, cysteine; Val, valine; Met, methionine; Leu, leucine; Phe, phenylalanine; GABA, gamma amino butyric acid; Lys, lysine; His, histidine; Arg, arginine; Pro, proline; Tyr, tyrosine; Ile, Isoleucine

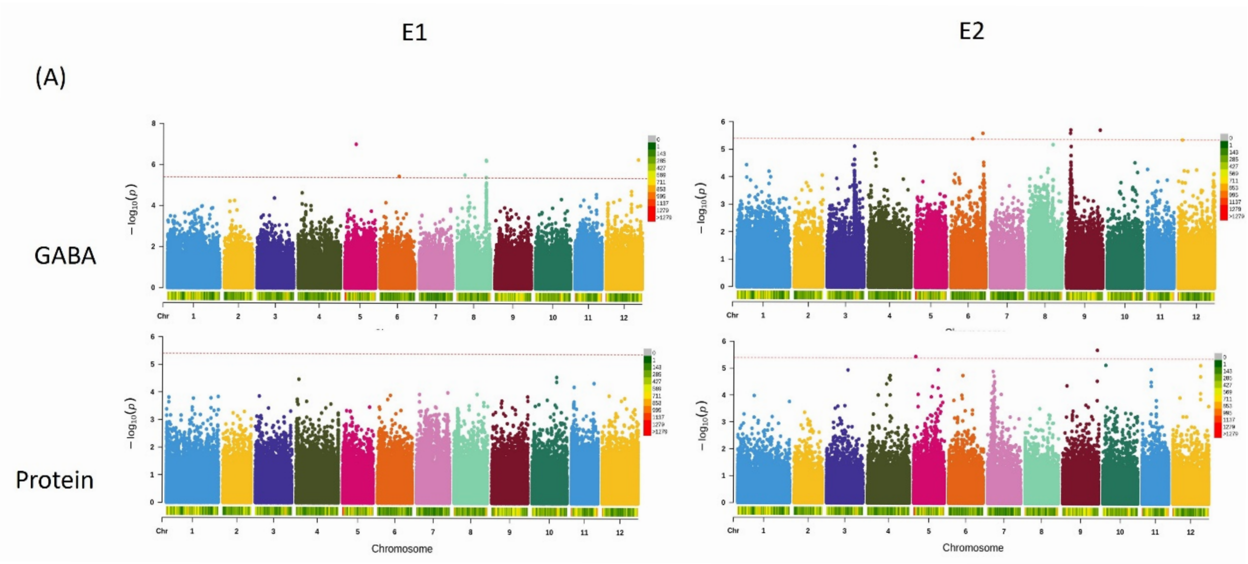
E1
E2
(A)
GABA
Protein
Chr
Chromosome
− log10(p)

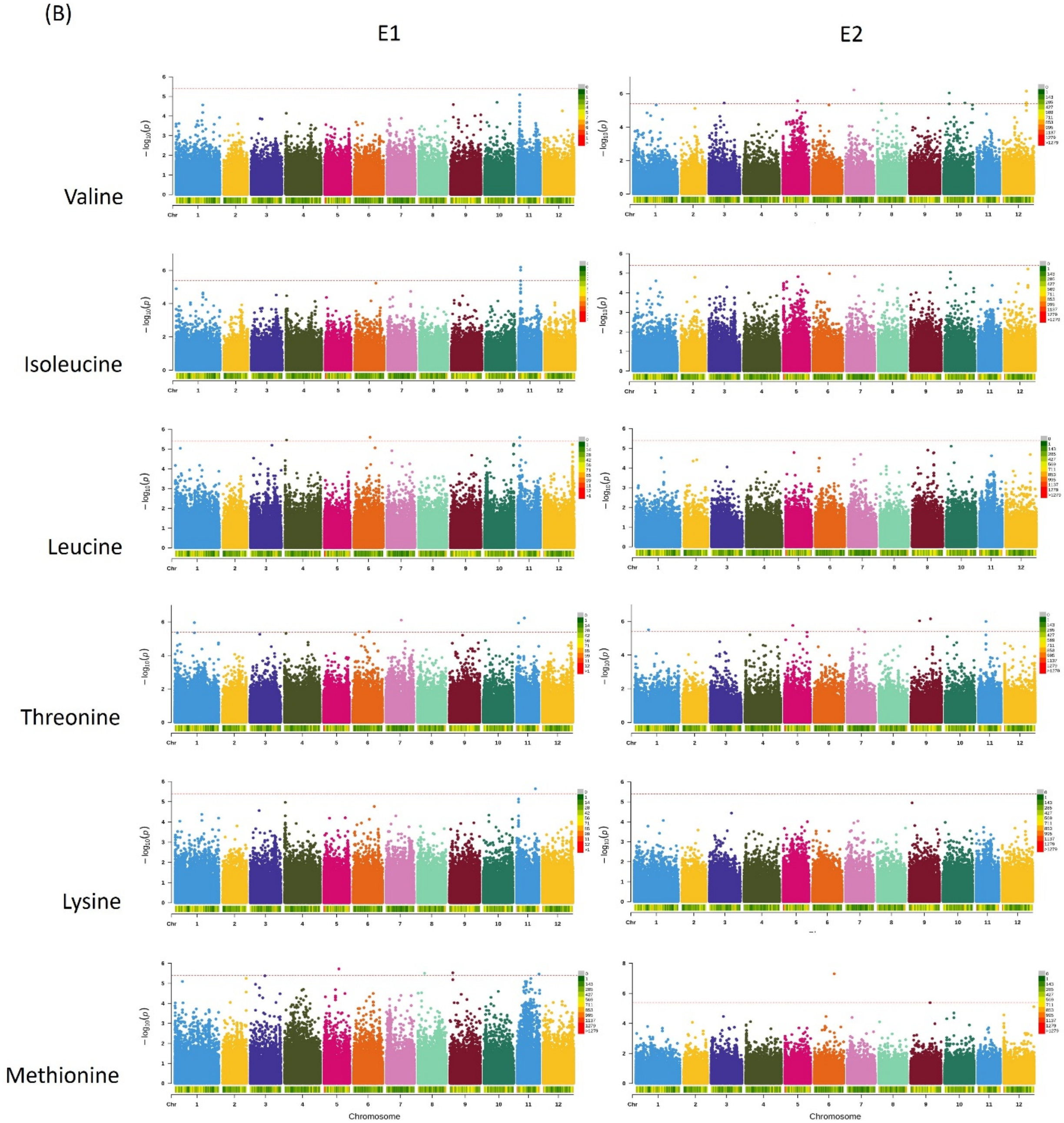
(B)
E1
E2
Valine
Isoleucine
Leucine
Threonine
Lysine
Methionine
$-\log_{10}(p)$
Chr
1 2 3 4 5 6 7 8 9 10 11 12
Chromosome

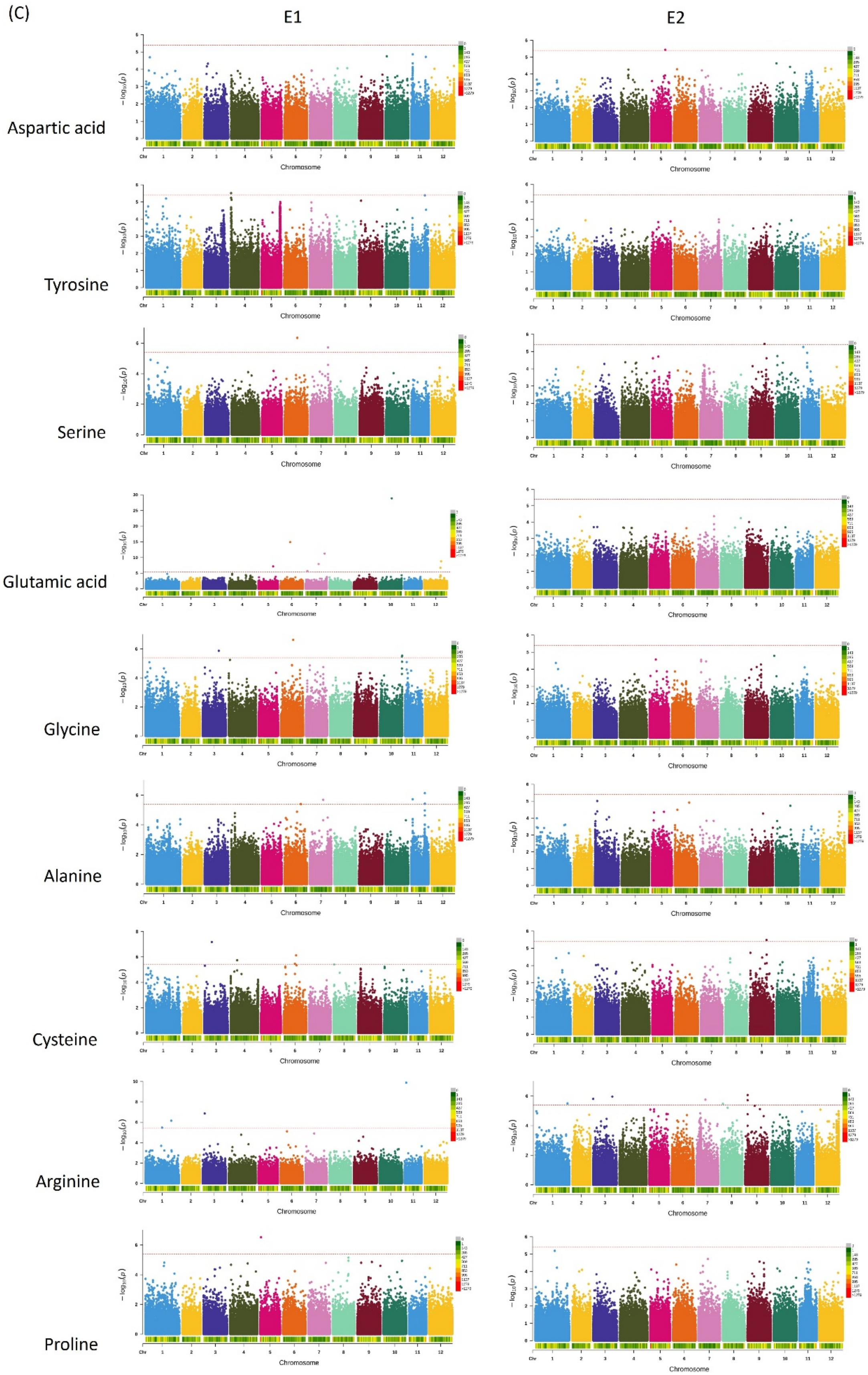
(C)
E1
E2
Aspartic acid
Tyrosine
Serine
Glutamic acid
Glycine
Alanine
Cysteine
Arginine
Proline
Chromosome

Figure S2. Genome-wide association study of protein and amino acid contents in potatoes across two environments. Manhattan plots represent marker traits associated with protein and amino acids. (**A)** represents GABA and Protein contents (**B)** represents essential amino acids, and (**C)** represents non-essential amino acids. E1 represents environment I, E2 represents environment II; Each dot represents an SNP. The x-axis represents chromosomal numbers and SNPs positions. The y-axis represents the negative logarithm p-value for individual SNP. The broken red line represents the significant threshold 4.02×10^{-6}. The heat maps at the bottom of each chromosome represent SNP density.

Table S1. List of potato accessions.

Code	Accession	Code	Accession
SP01	Kexin 2	SP53	Yunshu 401
SP02	Yunshu 102	SP54	Zhongshu 18
SP03	Yidunqing	SP55	Kexin 19
SP04	Longshu 5	SP56	Dongnong 303
SP05	Chuanliangshu 2	SP57	Gaoyuan 4
SP06	Zaodabai	SP58	Yunshu 301
SP07	Yunshu 302	SP59	Hui 2
SP08	Zhongshu 1	SP60	Yumalingshu 1
SP09	Deshu 2	SP61	Qing 168
SP10	Minshu 1	SP62	Jizhangshu 14
SP11	Qitaibaiyu	SP63	Da92062 1
SP12	Kexin 13	SP64	Chunshu 4
SP13	Zhongshu 2	SP65	Jizhangshu 8
SP14	Yunshu 503	SP66	Longshu 7
SP15	Zhengshu 6	SP67	Hushu 1
SP16	Chuanliangshu 8	SP68	Neinongshu 1
SP17	Dongnong 308	SP69	Zhongshu 20
SP18	Chuanyuzao	SP70	Longshu 6
SP19	Yunshu 101	SP71	Youjin
SP20	Zhongshu 7	SP72	Gaoyuan 7
SP21	Zhengshu 5	SP73	Zhongshu 5
SP22	Jinguan	SP74	Zhuanxinwu
SP23	Zhongshu 10	SP75	Shirenguo
SP24	Kexin 22	SP76	Kexin 12
SP25	Shaza 15	SP77	Chuanyu 10
SP26	Qingshu 8	SP78	Weiyu 3

SP27	Kexin 18	SP79	Yunshu 103
SP28	Neishu 7	SP80	Hongyu
SP29	Hezuo 88	SP81	Daxiyang
SP30	Qingshu 6	SP82	Heimeiren
SP31	Jinshu 16	SP83	White potato
SP32	Yunshu 505	SP84	Baked potato
SP33	Xishu 1	SP85	Red potato
SP34	Liangshu 97	SP86	Lijiang
SP35	Katading	SP87	Zhongshu 3
SP36	Jizhangshu 12	SP88	Zhongshu 6
SP37	Ningshu 6	SP89	Favorita
SP38	Qingshu 9	SP90	Xinjia 2
SP39	Jinkengbai	SP91	Bashu
SP40	Weiyu 5	SP92	Landrace (Jiande)
SP41	Keyi	SP93	Landrace (Hangzhou)
SP42	Heishanyao	SP94	Landrace (Lishui)
SP43	Lishu 7	SP95	Hucunyangyu
SP44	Chunshu 2	SP96	Dayangyangyu
SP45	Hongmei	SP97	Landrace (Dongyang)
SP46	Yanshu 4	SP98	Dongcang
SP47	Dongnong 311	SP99	Sanmengxiaohuangzhong
SP48	Mila	SP100	Landrace (Chun'an)
SP49	Xindaping	SP101	Landrace(pan'an) black
SP50	Yunshu 501	SP102	Landrace(pan'an) red
SP51	Jizhangshu 8	SP103	Landrance (Pan'an) white
SP52	Lishu 6	SP104	Xisen3
